# Antimicrobial, function, and crystalline analysis on the cellulose fibre extracted from the banana tree trunks

**DOI:** 10.1038/s41598-023-42160-8

**Published:** 2023-09-15

**Authors:** Raja Thandavamoorthy, Yuvarajan Devarajan, Nandagopal Kaliappan

**Affiliations:** 1 Material Science Lab, Department of Prosthodontics, Saveetha Dental College and Hospitals, SIMATS, Chennai, Tamilnadu India; 2 Department of Mechanical Engineering, Saveetha School of Engineering, SIMATS, Chennai, Tamilnadu India; 3 Department of Mechanical Engineering, Haramaya Institute of Technology, Haramaya University, Dire Dawa, Ethiopia

**Keywords:** Environmental sciences, Engineering, Biomedical engineering, Mechanical engineering

## Abstract

Bioactive substances such as phenolic compounds, antioxidants, and antibacterial agents are found in natural fibres. In this study, banana fibre was extracted from the trunks of banana plants. Antibacterial activity, FTIR, XRD, and SEM analysis were performed to characterize the banana cellulose fibre, and also raw and alkali-treated banana fibre composite was fabricated with an epoxy matrix. Results of the antibacterial analysis indicate that this banana cellulose fibre strongly impedes bacterial growth with elevated inhibitory zones. The primary peaks observed at 1170 cm^−1^ and 1426 cm^−1^ by FTIR analysis correspond to C–O stretching, O–H bending, aliphatic ether, secondary alcohol, and carboxylic acid. The morphological analysis reveals the fibre quality, and the EDX analysis confirms the elements present in the banana cellulose fibre. The XRD results demonstrated a more significant proportion (76.8%) of the amorphous region. This study indicates that banana cellulose fibre could be a promising source of antimicrobial compounds. In addition, the mechanical properties of alkali-treated banana fibre composite were preferable to raw fibre composite by an average of 3% for this banana fibre composite. As a result, this composite can be used to manufacture automobile interior components, as it can reduce the sanitizing periods of interior components during winter months.

## Introduction

Natural fibres can suppress bacterial development, a significant benefit in various applications. Furthermore, these fibres are biocompatible and biodegradable, which are important for biomedical materials to minimize difficulties owing to infections and severe immune reactions^1^. As a result, tissue engineering, medical textiles, orthopaedics, dental implants, and cosmetics are all extending their usage of plant fibres^2^. This review includes the sources of natural fibres with antibacterial characteristics, antimicrobial mechanisms, and biological uses. Banana tree peels and leaves have antioxidant characteristics and biological functions such as anti-diabetic, anti-diarrheal, anti-tumour, anti-mutagenic, and anti-ulcerogenic capabilities^3^. Fabrics crafted from linen, merino wool, and hemp have inherent antibacterial properties. Gram-negative *Escherichia coli* and Gram-positive *Staphylococcus* *aureus* were tested for antibacterial activity. Soil burial testing looked into how quickly certain fibre mixtures biodegrade^4^. The findings demonstrate that individual CLY fibres have potent antibacterial activity against *E*. *coli* and *S*. *aureus*. Antibacterial refers to any substance that kills bacteria or prevents them from multiplying^5^. Antibiotics, heat, and chemicals like chlorine may all kill germs. Nowadays, you can get a lot of antibacterial cleaning supplies and soaps in stores. Due to the abundance of substrates, it provides for fermentation events carried out by certain species of microbe that contain the enzymatic machinery to digest these complex carbohydrates; dietary fibre can profoundly affect the composition, variety, and richness of the microbiome^6^. The banana plant is extremely popular and has many practical applications. This plant's fruit, leaves, flower buds, trunk, and pseudostem are all edible^7^. The banana plant's pseudostem fibre is the topic of this section. The article explains the steps in growing, harvesting, retting, and degumming banana pseudostem fibre from the plant. Antimicrobial testing using the Agar plate method and the modified Hoenstein test utilized one gram-positive bacteria (*S*. *aureus*) and one gram-negative bacteria (*E*. *coli*)^8^. It was discovered that banana cloth treated with a cellulose enzyme had a 97% success rate against *S*. *aureus* and a 92% success rate against *E*. *coli*. Three washing cycles of the cellulose enzyme-treated banana fabric were shown to maintain its antibacterial properties^9^. On the other hand, untreated cloth lost its antibacterial finish after just one wash. The results showed that the antimicrobial property of the banana fabric could be improved by treating it with cellulose enzyme, which is an environmentally friendly approach to boosting surface characteristics and adsorption of the antimicrobial extracts. Not only does the surface of the cellulosic textile act as a transporter for bacteria, but it also serves as an outstanding growth substrate. Since the moisture recapture capacity of banana fibre is 15.8%, significantly greater than cotton's (8.5%), the banana fabric is more sensitive to developing microorganisms^10^. To determine how enzymatic degumming influences the integrity of hemp fibres, both untreated and treated hemp fibres were observed using SEM and fluorescent microscopy. This analysis was conducted to determine hemp fibres' protein content and quality and the efficacy of the enzymatic degumming process^11^. The chemical reactions of polymers (cellulosic compounds) are profoundly and intricately affected by cellulose structure. In general, cellulose has three structural levels the molecular level of a single macromolecule, the supra molecular level of packing and mutual ordering of the macromolecules, the morphological level of the architecture of already complex structural entities, and the corresponding pore system^12^. Some researchers have employed Fourier transform infrared spectroscopy (FTIR) in combination with mechanical loading to investigate the molecular reactions to stress and load, such as for spruce wood and cellulose paper materials, to get a better understanding of wood and wood fibres for their prospective application in advanced materials^13^. According to the standard two-phase cellulose model, cellulose chains comprise crystalline (ordered) and amorphous (less ordered) regions. The crystallinity index (CI) is a parameter used to characterize the relative quantity of crystalline material present in cellulose. This approach can only yield relative values since the spectrum always comprises contributions from crystalline and amorphous areas. The absorbance ratio A1420/A893 was defined as an empirical CI. The absorbance at 1420 cm^−1^ and 894 cm^−1^ in cellulose is sensitive to the amount of crystalline vs amorphous structure, implying that the widening of these bands suggests a more disordered structure. Numerous reports on TCI do not appear to show a consistent outcome^14^. Eco-friendly materials like tissue, filter paper, and currency paper can all be made from organic banana fibre. It is widely employed in the textile and apparel industries because of its strong tensile strength, heat resistance, good spinning ability, and natural origin. It's malleable enough to mix with various fibres^15^. The importance of biocomposite structures and the number of fields in which they can be used have grown steadily as people have become more aware of the need to protect the planet. Banana fibres are now recovered from agricultural by-products and reinforced in various composites. Banana fibres have several desirable characteristics: high strength, good dimensional stability and mechanical qualities, sensitivity to their surrounding environment, practicality, low cost, biodegradability, renewability, and sustainability^16^. Banana fibres are good for the planet and have other desirable properties, including low density, lightweight, low cost, excellent tensile strength, and resistance to water and fire. There are numerous potential construction and building material uses for this type of trash. Banana fibre is a by-product of the banana industry. Hence it can be used as a raw material in manufacturing at no cost^17^.

The above literature was used to start this research work, banana cellulose fibre extracted from their trunk. Further, to analyze the antimicrobial, functional, crystalline effect, and micrograph analysis, the mechanical effect of banana fibre (raw fibre and alkali treated) to make a composite for automobile and biomedical instruments application.

## Materials and methods

This study complies with relevant institutional, national, and international guidelines and legislation. Go Green Pvt. Ltd. supplied the banana fibre and epoxy matrix in Chennai, India. The banana's stem is manually skinned in the banana fibre extraction apparatus. The brown and green outer bark is discarded after being peeled. Now the trunk's interior epidermis is white. This inner husk extracts the fibres from bananas: retting and mechanical extraction procedures separate plant fibres^18^. Typical retting techniques include water, condensation, chemical, and enzyme retting. The most common procedure for extracting high-quality fibres is water retting. The chemical composition of banana fibres is 73.92 per cent cellulose, 11.72 per cent hemicellulose, and 8.23 per cent lignin, with a diameter of 138 mm and a density of 1.59 g/cm^3^^19^. It avoids common issues associated with the traditional filtration method using frits and filter beds, facilitates sample handling and digestion, and ensures more accurate and precise analysis results^20^.

**Figure 1:**
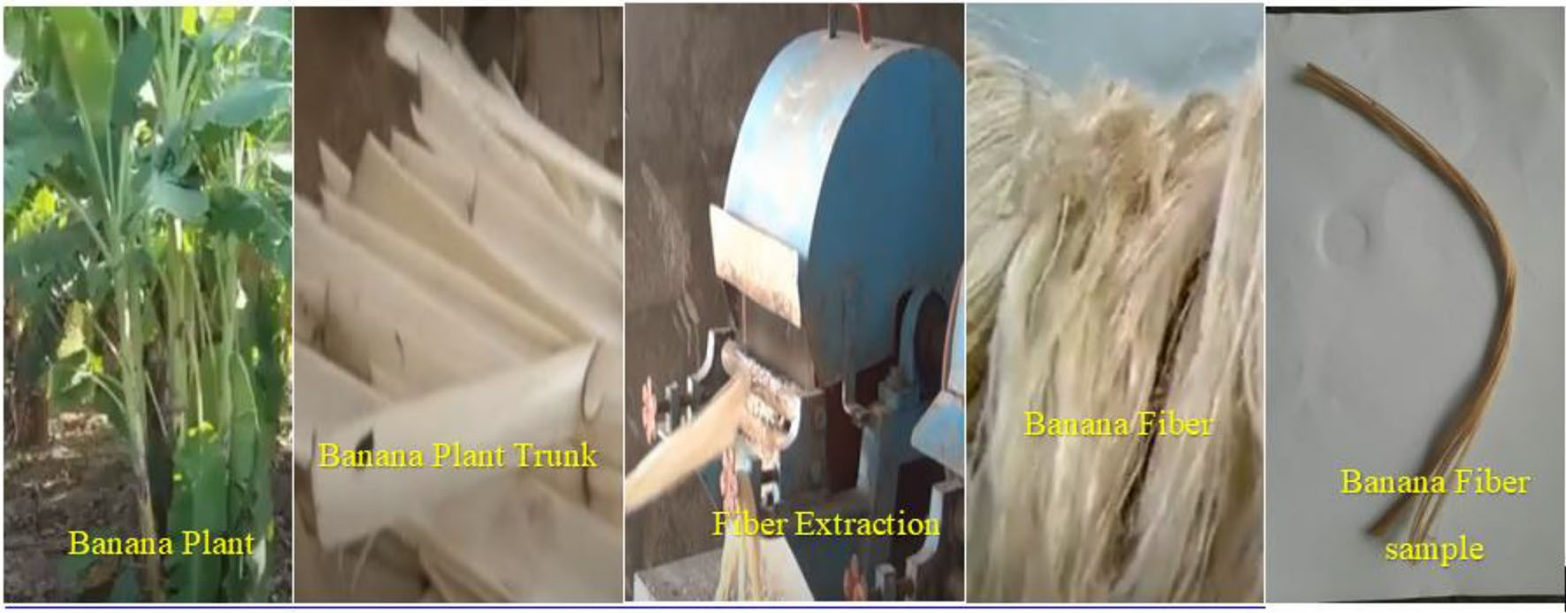
The process of fibre extraction from banana plant trunks.

A machine with pressure rollers and a fibre separation mechanism are used to treat the white section (inner bark) that has been removed. The white inner bark retains a great deal of moisture and must be removed before the tree can be harvested. To remove excess moisture from fibres, pressure rollers are used. The peeled white bark is passed between nippy rollers to remove excess moisture^21^. Drying out is like pressing sugarcane to get the juice out. The fibres are given a thorough washing in hot water after that. The fibres are compressed and hung from the wooden railing to dry in the sun. After the fibre has dried thoroughly, it can be utilized in further experiments. The fibre extraction process from banana plant trunks is shown in Fig. 1. Then the composite laminates were fabricated from extracted raw fibre and alkali-treated banana fibre with 50%, and another 50% epoxy matrix was used to make the natural fibre composite underhand layup technique^22^.

### Experimental testing of banana fibre

An extract or pure chemical's in vitro antibacterial activity may be evaluated or screened using several laboratory techniques. Disk diffusion and broth or agar dilution are the most common and fundamental techniques. The latter was utilized for this study. A spectrum scan of infrared-absorbing substances may be created using Fourier Transform Infrared Spectrometry (FTIR) according to ASTM E168. Banana fibre X-Ray Diffraction Phase Proportion Standard Test Method C1365, American Society for Testing and Materials^23^. To reliably measure the diameter of an electron beam in a scanning electron microscope, it is recommended to follow the procedures outlined in ASTM E986. Banana fibre can be analyzed for the presence of elements using an Energy Dispersive X-ray (EDX) microanalysis technique, an elemental analysis method related to electron microscopy based on creating distinctive X-rays^24^. Then the banana fibre was tested under mechanical tests such as tensile strength (ASTM D638), flexural strength (ASTM D790), and Izod impact energy absorption (ASTM D256) of raw banana fibre and alkali-treated banana fibre composite^25^.

## Results and discussion

The potential of banana fibre, which is extracted from the banana plant's trunk, as a renewable and sustainable material has increased its profile in recent years. This work briefs about antibacterial analyses and the possible uses of natural fibres, while little research is devoted to the antibacterial characteristics of banana fibre.

### Antibacterial properties of banana cellulose fibre

**Figure 2:**
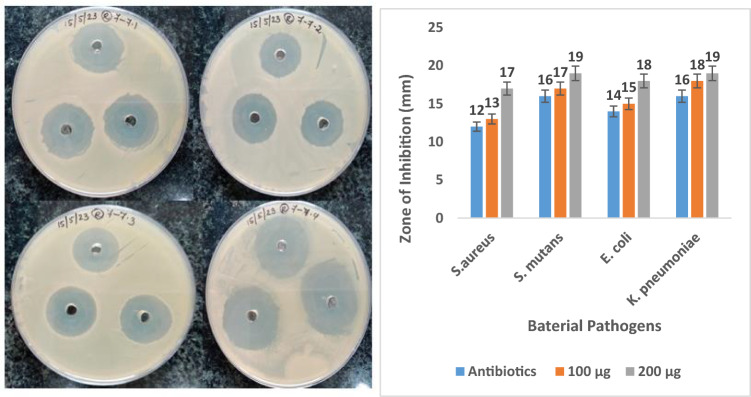
Antibacterial inhibition zone of banana cellulose fibre.

The most frequent bacteria detected in biomedical device infections are taken for this work. They are *S*. *aureus*, *Streptococcus mutans*, *E*. *coli*, and *Klebsiella pneumonia*^26^. The antibacterial inhibition zone of banana cellulose fibre is shown in Fig. 2.

Infectious gram-positive bacteria such as *S. aureus* and *S. mutans* and gram-negative bacteria such as *E. coli* and *K. pneumonia* were cultured in nutrient broth flasks with all the needed nutrients to grow. Plates of Agar were inoculated with bacterial test suspensions (15 μg) containing 100 cells ml^−1^. Natural chemicals, such as phenolic compounds released by some fibres, have antimicrobial effects. Bacteria can be killed or stopped in their tracks by the rough surface of natural fibres. One mechanism by which some fibres exert antimicrobial effects is by soaking up bacterial molecules like toxins. The well was then stuffed with 100 μg and 200 μg of banana cellulose fibre sample. Then, identify the effectiveness of conventional antibiotics on gram-positive and gram-negative bacteria. The samples were incubated at 5 °C for 15 min to allow for diffusion and then at 32 °C for 48 h to allow for bacterial growth^27^. The results revealed banana cellulose fibre contains antibacterial activities observed in the formation of inhibition zone in the agar plate. Therefore, the test was considered successful after the incubation of an inhibitory zone formed around the well. The antibacterial capabilities of banana fibre have been well studied. Banana natural fibres have shown antibacterial activity. Methods now used to combat bacterial infections in biomedical devices, and implants aim to either reduce bacterial adherence on their surfaces or destroy the germs already attached to the device or implant. Most commonly, plant fibres are altered to display two key features. The first feature is a bactericidal action, which results in the death of bacteria by introducing a bioactive molecule that disrupts the cytoplasmic membrane, alters the membrane conductivity, inhibits protein synthesis, and inhibits the production of nucleic acids^28^. The data suggests that banana fibre could have comparable qualities. Banana fibres show the antibacterial effect of inhibition and are increasingly used in textiles, including beds, clothes, and towels, to combat the spread of bacteria, germs, and unpleasant odors^29^. Wound dressings, surgical sutures, and other medical textiles can benefit from antibacterial fibres, which kill germs and facilitate healing^30^.

### Fourier transform infrared (FTIR) spectroscopy analysis

To determine the chemical makeup of a substance, such as cellulose fibres, Fourier Transform Infrared (FTIR) spectroscopy is frequently employed. FTIR can reveal the presence of functional groups and molecular structures in a substance by analyzing the sample's absorption of infrared light^31^. This paper reviews how FTIR analysis may be used to investigate banana cellulose fibre. A spectrometer and infrared light source make up the FTIR device. The range of infrared frequencies from the light source is 4000–400 cm^−1^^32^. The spectrometer records how much light of different frequencies is absorbed by the sample during transmission. The FTIR spectrum produced may then be used to identify and characterize the functional groups in the material. Typical peaks associated with cellulose and other components found in banana cellulose fibre may be seen in the FTIR spectrum of the fibre. One must compare the detected peaks to reference spectra or databases to deduce the functional groups and chemical bonds. O–H stretching at 3330–3400 cm^−1^, C–H stretching at 2900–3000 cm^−1^, C=O stretching of absorbed water at 1650 cm^−1^, nd C–O–C stretching of glycosidic links at 1050–1150 cm^−1^ are the most prominent peaks in a typical cellulose FTIR spectrum^33^.

**Figure 3:**
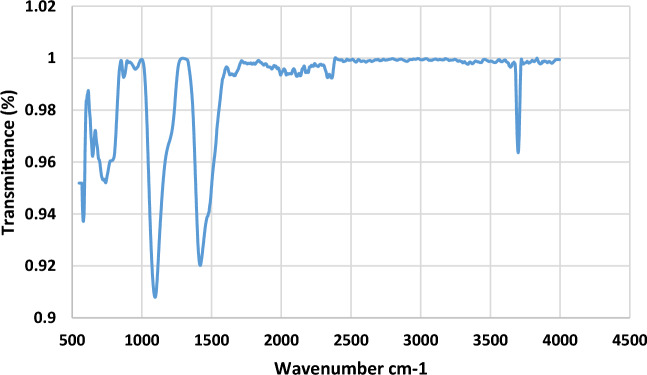
FTIR analysis of banana cellulose fibre.

Figure 3 displays the results of the FTIR analysis. Impurities, hemicellulose, lignin, and other additions to the banana cellulose fibre may also appear as peaks in the spectrum. The unprocessed stems of select textile plants were qualitatively characterized in advance using a feasible and minimally intrusive method that did not require extensive extraction operations. Flax (*Linum* *usitatissimum* L.), velvet leaf (*Abutilon* *theophrasti* Medik.), hemp (*Cannabis* *sativa* L.), and jute (*Corchorus* *olitorius* L.) stems were subjected to Fourier Transform Infrared Spectroscopy (FT-IR) comparisons in search of quality markers. The primary components were shown to be present in the analyzed plants' stems by analysis. The crystalline area of cellulose corresponds to a band at 1420–1428 cm^−1^, and the amorphous region of cellulose corresponds to a band at 896–898 cm^−1^^34^. These peaks can provide details about the fibre's composition and any alterations made by this banana cellulose fibre. Anti-symmetric stretching of C–O–C groups was verified in hemicellulose and cellulose molecules, as evidenced by a peak at 1160 cm^−1^. The polysaccharide –CH2 wagging vibration is responsible for the peak at 1318 cm^−1^. The lignocellulose fibres were modified using a polypropylene–maleic anhydride copolymer. In another research work, the natural fibre extracted from the hemp plant and analyzed their functional group under untreated hemp fibre and alkali-treated hemp fibre. The findings show that the untreated hemp fibres' spectra varied from the treated hemp fibres' spectrum by two peaks, one at 1739 cm^−1^ and one at 1746 cm^−1^ ^35^. According to the FTIR study, there are two main processes in the reaction between fibres and copolymers: first, the copolymer is changed into the more reactive anhydride form, and then esterification occurs on the surface of cellulose fibres^35^.

### XRD analysis of banana cellulose fibre

X-ray diffraction (XRD) analysis effectively determines the crystalline structure of materials such as cellulose fibres by exposing the sample to X-rays and analyzing the resulting diffraction pattern^36^. XRD provides information about the arrangement of atoms in the material and can provide insight into its crystal structure. Here is a summary of how XRD analysis can investigate banana cellulose fibre. For XRD analysis, banana cellulose fibres must be produced as a narrow, uniform sample. This can be accomplished by crushing the fibres into a fine powder or mounting them as a thin film or solid sample on an appropriate substrate. Typically, the X-ray source emanates X-rays with a fixed wavelength interacting with the sample. The detector then collects the diffracted X-rays and records the resulting diffraction pattern. Crystal structure peaks in banana cellulose fibre's XRD diffraction pattern indicate this. Understanding the pattern's structure requires looking at the location, magnitude, and shape of the pattern's peaks. The (002) peak, representing the distance between cellulose chains in the crystal lattice, stands out most clearly in an XRD pattern of cellulose fibres. It usually shows up between 2*θ* = 22 and 24 degrees. The crystallinity index, which indicates how much fibre is composed of the crystalline phase, may be determined from the XRD pattern^36^.

**Figure 4:**
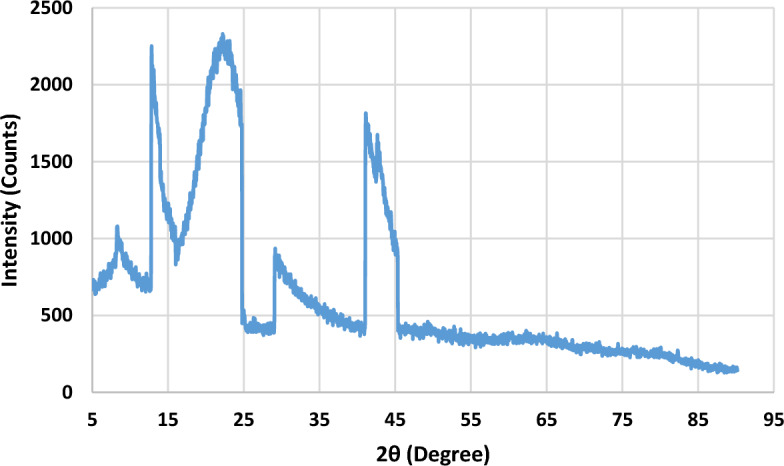
XRD analysis of banana cellulose fibre.

The peaks observed from this analysis 2theta value at 14, 23, and 37. The corresponding intensities are 2329, 2380, and 1730 counts, respectively. From this analysis, the crystalline effect reveals the banana cellulose fibre contains 86.9% amorphous region and 13.1% crystal region. In a separate study, sisal fibre reveals a crystalline size of 52 nm and a crystallinity index of 61.2%, with peaks observed at 24, 36, and 38 degrees of 2 theta values, which is used to indicate a 24% crystalline effect and a 76% amorphous region because natural fibres contain a greater proportion of amorphous regions^37^. Therefore, this banana cellulose fibre can reinforce the polymer composite material. The XRD curve of banana cellulose fibre is shown in Fig. 4.

### SEM analysis of banana cellulose fibre

**Figure 5:**
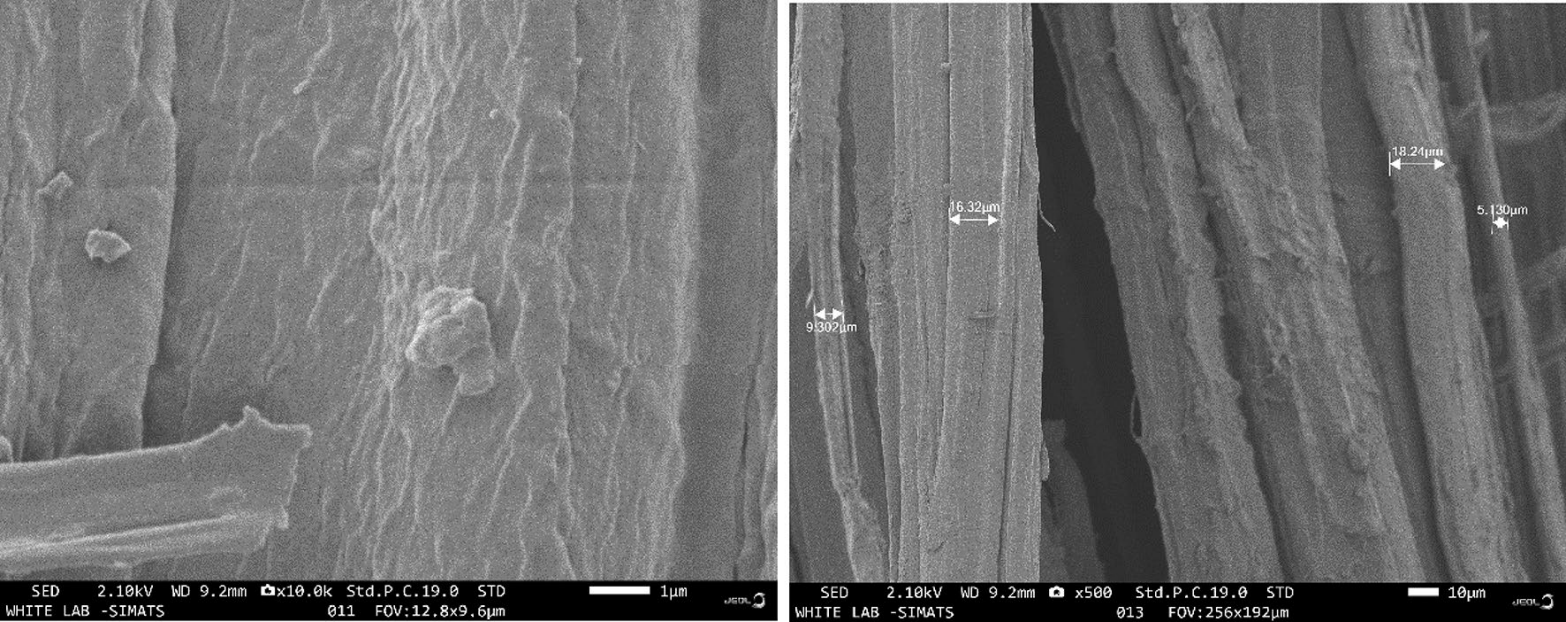
SEM image of banana fibre.

**Figure 6:**
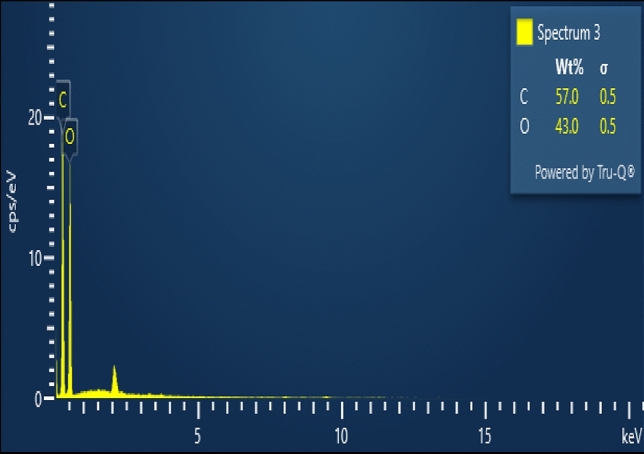
EDX analysis of banana cellulose fibre.

Using low-voltage Scanning Electron Microscopy (LV-SEM), the microstructure of several natural plant fibres were characterized. Banana and sisal fibres were shown to have less variation in cross-sectional area, internal lumen form and size, and cell wall thickness than flax and ramie fibres, as observed by LV-SEM^12^. Banana fibre may be analyzed for its surface shape and elemental composition using scanning electron microscopy (SEM) in conjunction with energy-dispersive X-ray spectroscopy (EDX). This enables the high-magnification visualization of the fibre's microstructure and the determination of the elemental composition of regions of interest. After the sample of banana fibre has been produced, it is fed into the SEM and subjected to an electron beam. The natural fibre is derived from the banana tree's stem, is biodegradable, and extremely resilient. The fibre mostly comprises cellulose, hemicelluloses, and lignin and comprises thick-walled cell tissue held together by natural gums^16^. The diameter of the fibre, its surface characteristics, and structural defects are all shown in Fig. 5. For several reasons, including growth circumstances and fibre treatments, carbon, oxygen, hydrogen, and other trace elements may be present in the banana fibre. It can be revealed by EDX analysis, as shown in Fig. 6.

### Mechanical properties of banana trunk fibre composite

**Figure 7:**
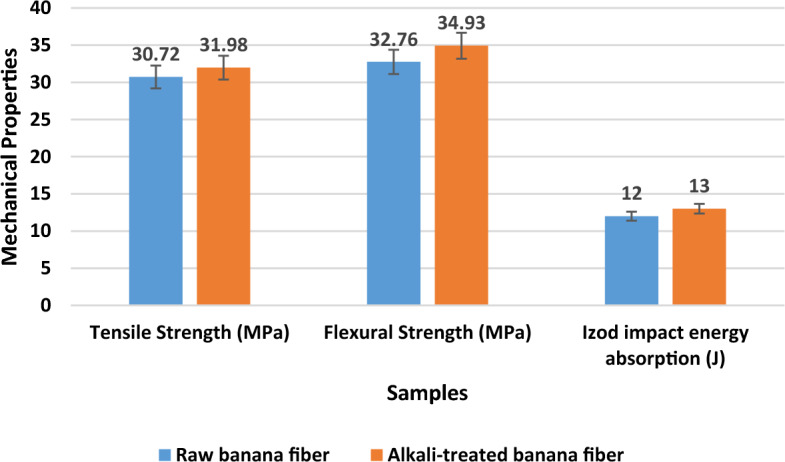
Mechanical properties of banana fibre composite.

The composite material is manufactured from unprocessed banana fibre and fibre treated with an alkali. Figure 7 depicts the mechanical properties of banana fibre composite. 100 g chopped banana fibre (raw and alkali treated) and 100 g epoxy matrix were used to fabricate the composite, and the results demonstrate the superior tensile strength (31.98 MPa), flexural strength (34.93 MPa), and impact energy absorption capacity (13 J) of this banana fibre as a result of the alkali treatment process.

Similar work was performed with flax fibre composite, and the results were found to be 2.6% superior due to alkali treatment on the flax fibre, which disclosed the removal of hemicellulose, reduction of lignin, and pectin content used to enhance the bonding strength of the natural fibre composite ^19^. Therefore, after banana fiber extraction, a chemical treatment is required to enhance the polymer composite's bonding strength.

## Conclusion

This research investigated the antibacterial capacity of natural fibre extracted from banana plant trunks. Banana cellulose fibre containing a significant bacterial inhibit zone was observed from this analysis under different micrograms. Further, the characterization work also reveals that the functional group of carbon and oxygen (C–O–C) was noted at peaks between 1000 and 1500 cm^−1^ and that the amorphous region represents the banana fibre that can be used as reinforcement for polymer composite materials. The FTIR data show that the chemical bonds between banana fibres are strong due to components including lignin, pectin, wax and hemicellulose. The morphological and elemental analysis confirmed that the extracted fibre layers strengthened the materials due to the fibre continuity and less damage to the fibre. Also, the carbon and oxygen show that natural banana fibre exhibits environmentally friendly materials used for biomedical applications. The natural composite (banana tree trunk is a waste product, so it converts to a useful product under sustainable development) antibacterial activity is used to reduce the sanitizing of parts. Also, the mechanical properties are superior in banana fibre composite used in automobile interior applications.

### Future scope of this study

This banana fibre can be used as reinforcement with other natural fibres to enhance its unique properties and create products based on a polymer composite reinforced with natural fibres. In addition, analyze the thermal properties and dynamical behaviours of composite materials containing hybridized banana and other natural fibres.

## Data Availability

The datasets used and/or analyzed during the current study available from the corresponding author on reasonable request.
